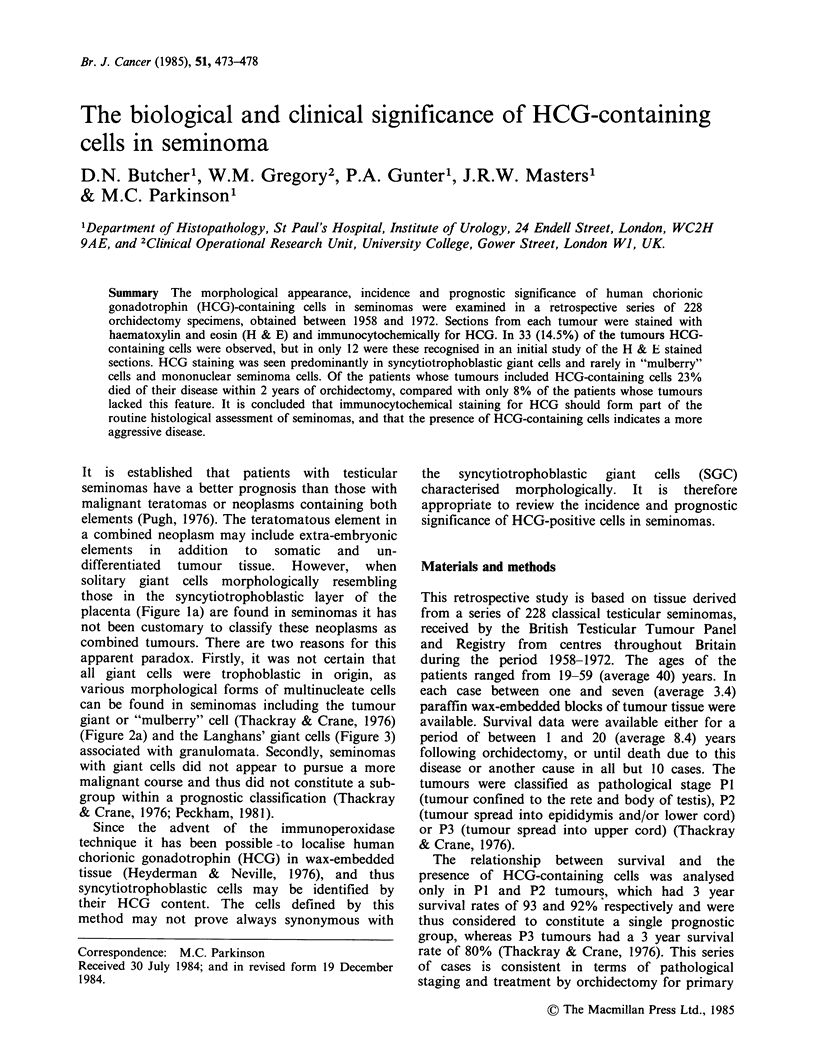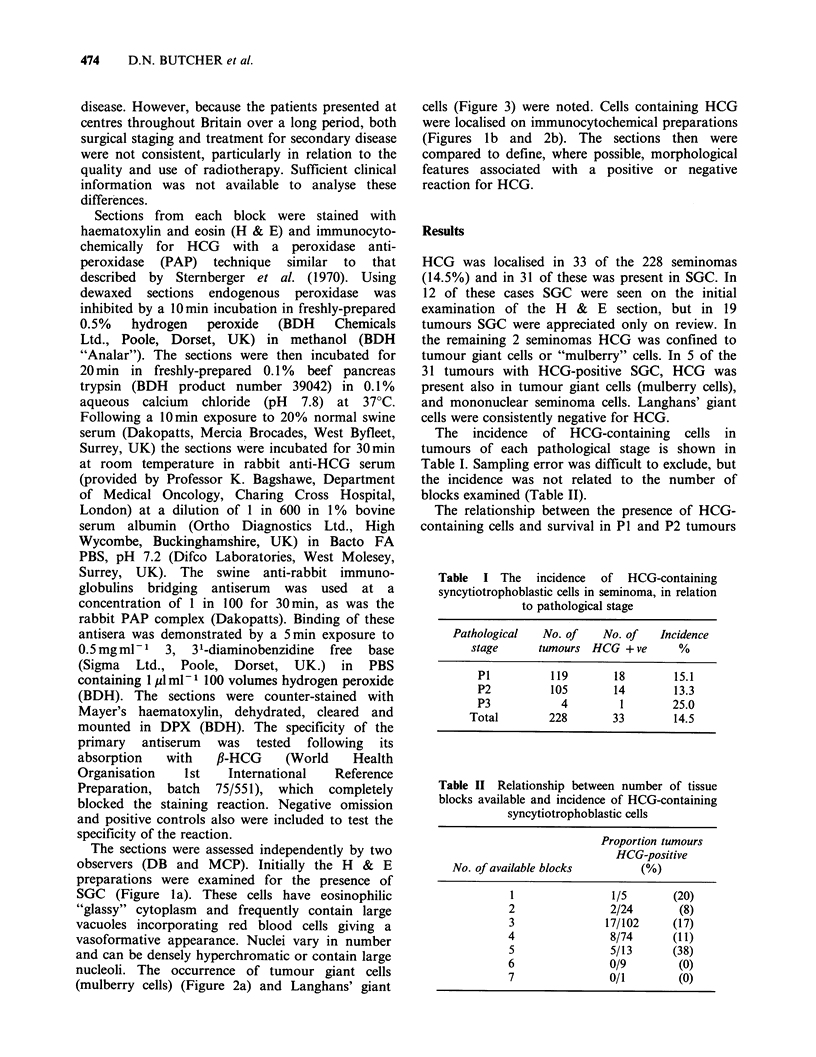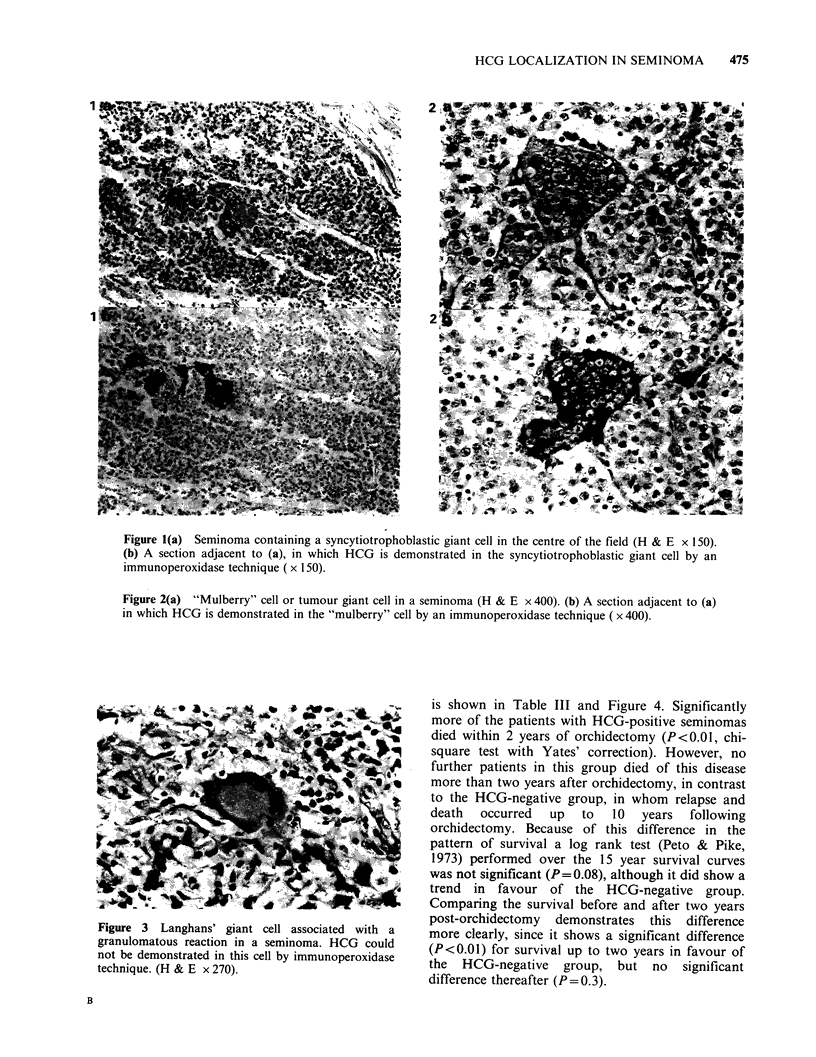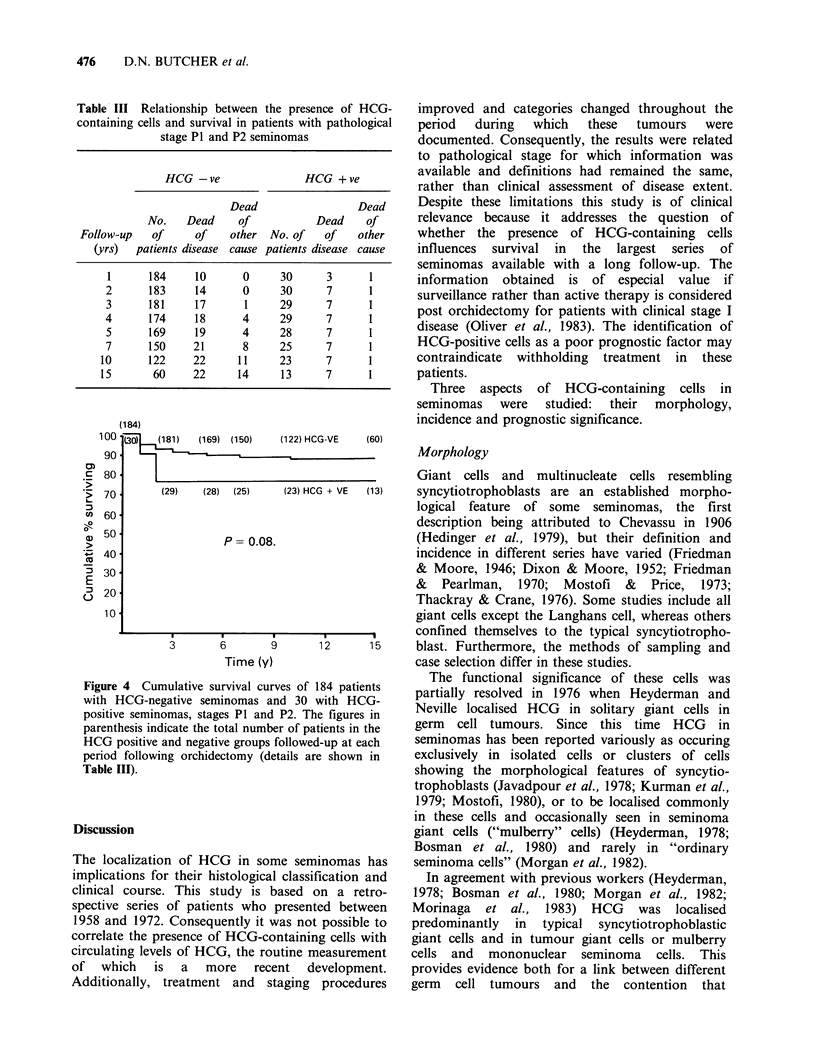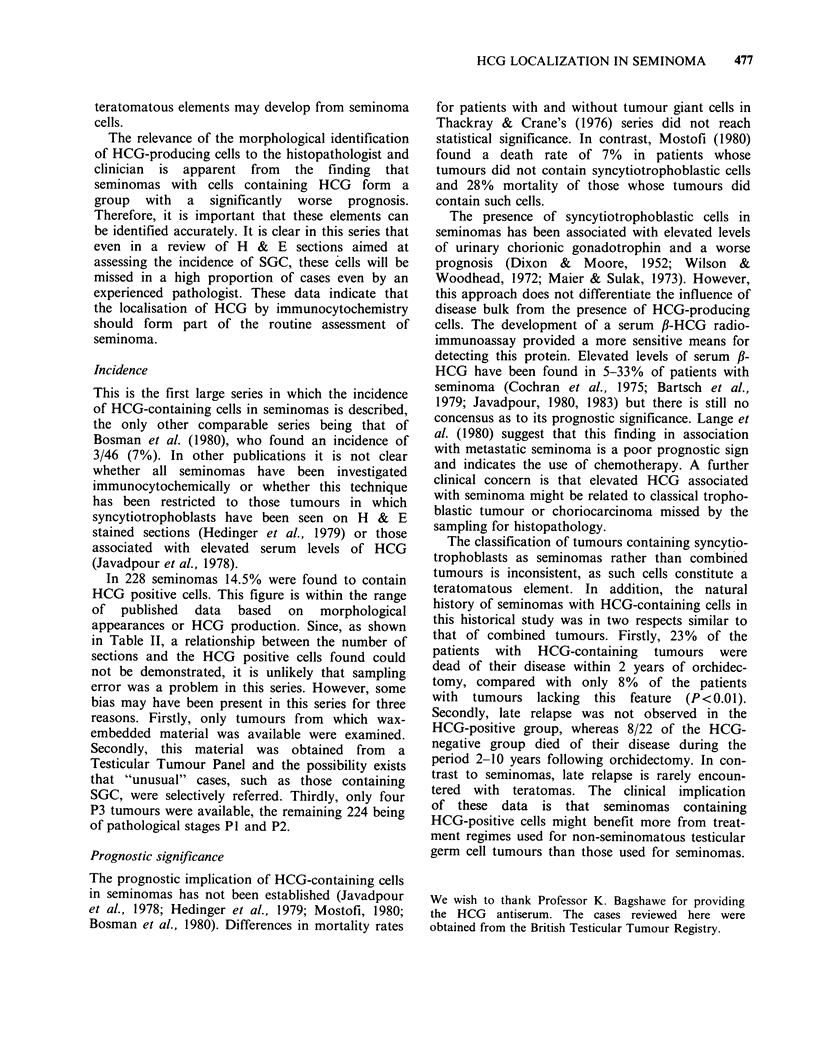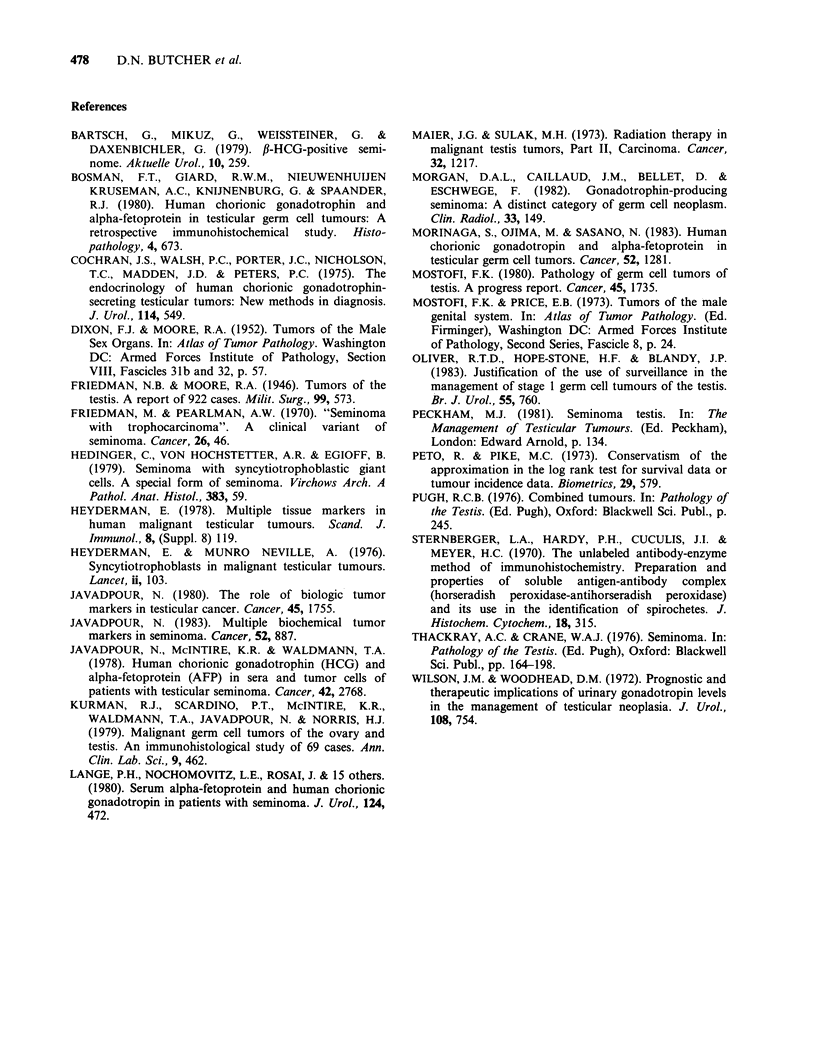# The biological and clinical significance of HCG-containing cells in seminoma.

**DOI:** 10.1038/bjc.1985.68

**Published:** 1985-04

**Authors:** D. N. Butcher, W. M. Gregory, P. A. Gunter, J. R. Masters, M. C. Parkinson

## Abstract

**Images:**


*Br. J. Cancer* (1985), **51**, 473–478

### The biological and clinical significance of HCG-containing cells in seminoma

D.N. Butcher[1], W.M. Gregory[2], P.A. Gunter[1], J.R.W. Masters[1] & M.C. Parkinson[1]

[1]*Department of Histopathology, St Paul's Hospital, Institute of Urology, 24 Endell Street, London, WC2H 9AE, and* [2]*Clinical Operational Research Unit, University College, Gower Street, London W1, UK.*

**Summary** The morphological appearance, incidence and prognostic significance of human chorionic gonadotrophin (HCG)-containing cells in seminomas were examined in a retrospective series of 228 orchidectomy specimens, obtained between 1958 and 1972. Sections from each tumour were stained with haematoxylin and eosin (H & E) and immunocytochemically for HCG. In 33 (14.5%) of the tumours HCG-containing cells were observed, but in only 12 were these recognised in an initial study of the H & E stained sections. HCG staining was seen predominantly in syncytiotrophoblastic giant cells and rarely in "mulberry" cells and mononuclear seminoma cells. Of the patients whose tumours included HCG-containing cells 23% died of their disease within 2 years of orchidectomy, compared with only 8% of the patients whose tumours lacked this feature. It is concluded that immunocytochemical staining for HCG should form part of the routine histological assessment of seminomas, and that the presence of HCG-containing cells indicates a more aggressive disease.

It is established that patients with testicular seminomas have a better prognosis than those with malignant teratomas or neoplasms containing both elements (Pugh, 1976). The teratomatous element in a combined neoplasm may include extra-embryonic elements in addition to somatic and undifferentiated tumour tissue. However, when solitary giant cells morphologically resembling those in the syncytiotrophoblastic layer of the placenta (Figure 1a) are found in seminomas it has not been customary to classify these neoplasms as combined tumours. There are two reasons for this apparent paradox. Firstly, it was not certain that all giant cells were trophoblastic in origin, as various morphological forms of multinucleate cells can be found in seminomas including the tumour giant or "mulberry" cell (Thackray & Crane, 1976) (Figure 2a) and the Langhans' giant cells (Figure 3) associated with granulomata. Secondly, seminomas with giant cells did not appear to pursue a more malignant course and thus did not constitute a subgroup within a prognostic classification (Thackray & Crane, 1976; Peckham, 1981).

Since the advent of the immunoperoxidase technique it has been possible to localise human chorionic gonadotrophin (HCG) in wax-embedded tissue (Heyderman & Neville, 1976), and thus syncytiotrophoblastic cells may be identified by their HCG content. The cells defined by this method may not prove always synonymous with the syncytiotrophoblastic giant cells (SGC) characterised morphologically. It is therefore appropriate to review the incidence and prognostic significance of HCG-positive cells in seminomas.

Correspondence: M.C. Parkinson
Received 30 July 1984; and in revised form 19 December 1984.

### Materials and methods

This retrospective study is based on tissue derived from a series of 228 classical testicular seminomas, received by the British Testicular Tumour Panel and Registry from centres throughout Britain during the period 1958–1972. The ages of the patients ranged from 19–59 (average 40) years. In each case between one and seven (average 3.4) paraffin wax-embedded blocks of tumour tissue were available. Survival data were available either for a period of between 1 and 20 (average 8.4) years following orchidectomy, or until death due to this disease or another cause in all but 10 cases. The tumours were classified as pathological stage P1 (tumour confined to the rete and body of testis), P2 (tumour spread into epididymis and/or lower cord) or P3 (tumour spread into upper cord) (Thackray & Crane, 1976).

The relationship between survival and the presence of HCG-containing cells was analysed only in P1 and P2 tumours, which had 3 year survival rates of 93 and 92% respectively and were thus considered to constitute a single prognostic group, whereas P3 tumours had a 3 year survival rate of 80% (Thackray & Crane, 1976). This series of cases is consistent in terms of pathological staging and treatment by orchidectomy for primary

© The Macmillan Press Ltd., 1985

disease. However, because the patients presented at centres throughout Britain over a long period, both surgical staging and treatment for secondary disease were not consistent, particularly in relation to the quality and use of radiotherapy. Sufficient clinical information was not available to analyse these differences.

Sections from each block were stained with haematoxylin and eosin (H & E) and immunocytochemically for HCG with a peroxidase anti-peroxidase (PAP) technique similar to that described by Sternberger *et al.* (1970). Using dewaxed sections endogenous peroxidase was inhibited by a 10 min incubation in freshly-prepared 0.5% hydrogen peroxide (BDH Chemicals Ltd., Poole, Dorset, UK) in methanol (BDH "Analar"). The sections were then incubated for 20 min in freshly-prepared 0.1% beef pancreas trypsin (BDH product number 39042) in 0.1% aqueous calcium chloride (pH 7.8) at 37°C. Following a 10 min exposure to 20% normal swine serum (Dakopatts, Mercia Brocades, West Byfleet, Surrey, UK) the sections were incubated for 30 min at room temperature in rabbit anti-HCG serum (provided by Professor K. Bagshawe, Department of Medical Oncology, Charing Cross Hospital, London) at a dilution of 1 in 600 in 1% bovine serum albumin (Ortho Diagnostics Ltd., High Wycombe, Buckinghamshire, UK) in Bacto FA PBS, pH 7.2 (Difco Laboratories, West Molesey, Surrey, UK). The swine anti-rabbit immunoglobulins bridging antiserum was used at a concentration of 1 in 100 for 30 min, as was the rabbit PAP complex (Dakopatts). Binding of these antisera was demonstrated by a 5 min exposure to $0.5\,mg\,ml^{-1}$ 3, $3^1$-diaminobenzidine free base (Sigma Ltd., Poole, Dorset, UK.) in PBS containing $1\,\mu l\,ml^{-1}$ 100 volumes hydrogen peroxide (BDH). The sections were counter-stained with Mayer's haematoxylin, dehydrated, cleared and mounted in DPX (BDH). The specificity of the primary antiserum was tested following its absorption with $\beta$-HCG (World Health Organisation 1st International Reference Preparation, batch 75/551), which completely blocked the staining reaction. Negative omission and positive controls also were included to test the specificity of the reaction.

The sections were assessed independently by two observers (DB and MCP). Initially the H & E preparations were examined for the presence of SGC (Figure 1a). These cells have eosinophilic "glassy" cytoplasm and frequently contain large vacuoles incorporating red blood cells giving a vasoformative appearance. Nuclei vary in number and can be densely hyperchromatic or contain large nucleoli. The occurrence of tumour giant cells (mulberry cells) (Figure 2a) and Langhans' giant cells (Figure 3) were noted. Cells containing HCG were localised on immunocytochemical preparations (Figures 1b and 2b). The sections then were compared to define, where possible, morphological features associated with a positive or negative reaction for HCG.

### Results

HCG was localised in 33 of the 228 seminomas (14.5%) and in 31 of these was present in SGC. In 12 of these cases SGC were seen on the initial examination of the H & E section, but in 19 tumours SGC were appreciated only on review. In the remaining 2 seminomas HCG was confined to tumour giant cells or "mulberry" cells. In 5 of the 31 tumours with HCG-positive SGC, HCG was present also in tumour giant cells (mulberry cells), and mononuclear seminoma cells. Langhans' giant cells were consistently negative for HCG.

The incidence of HCG-containing cells in tumours of each pathological stage is shown in Table I. Sampling error was difficult to exclude, but the incidence was not related to the number of blocks examined (Table II).

The relationship between the presence of HCG-containing cells and survival in P1 and P2 tumours

**Table I** The incidence of HCG-containing syncytiotrophoblastic cells in seminoma, in relation to pathological stage

| *Pathological stage* | *No. of tumours* | *No. of HCG +ve* | *Incidence %* |
|---|---|---|---|
| P1 | 119 | 18 | 15.1 |
| P2 | 105 | 14 | 13.3 |
| P3 | 4 | 1 | 25.0 |
| Total | 228 | 33 | 14.5 |

**Table II** Relationship between number of tissue blocks available and incidence of HCG-containing syncytiotrophoblastic cells

| *No. of available blocks* | *Proportion tumours HCG-positive (%)* | |
|---|---|---|
| 1 | 1/5 | (20) |
| 2 | 2/24 | (8) |
| 3 | 17/102 | (17) |
| 4 | 8/74 | (11) |
| 5 | 5/13 | (38) |
| 6 | 0/9 | (0) |
| 7 | 0/1 | (0) |

**Figure 1(a)** Seminoma containing a syncytiotrophoblastic giant cell in the centre of the field (H & E ×150). **(b)** A section adjacent to (**a**), in which HCG is demonstrated in the syncytiotrophoblastic giant cell by an immunoperoxidase technique ( ×150).

**Figure 2(a)** "Mulberry" cell or tumour giant cell in a seminoma (H & E ×400). (**b**) A section adjacent to (**a**) in which HCG is demonstrated in the "mulberry" cell by an immunoperoxidase technique ( ×400).

**Figure 3** Langhans' giant cell associated with a granulomatous reaction in a seminoma. HCG could not be demonstrated in this cell by immunoperoxidase technique. (H & E ×270).

is shown in Table III and Figure 4. Significantly more of the patients with HCG-positive seminomas died within 2 years of orchidectomy ($P<0.01$, chi-square test with Yates' correction). However, no further patients in this group died of this disease more than two years after orchidectomy, in contrast to the HCG-negative group, in whom relapse and death occurred up to 10 years following orchidectomy. Because of this difference in the pattern of survival a log rank test (Peto & Pike, 1973) performed over the 15 year survival curves was not significant ($P=0.08$), although it did show a trend in favour of the HCG-negative group. Comparing the survival before and after two years post-orchidectomy demonstrates this difference more clearly, since it shows a significant difference ($P<0.01$) for survival up to two years in favour of the HCG-negative group, but no significant difference thereafter ($P=0.3$).

**Table III** Relationship between the presence of HCG-containing cells and survival in patients with pathological stage P1 and P2 seminomas

| | *HCG −ve* | | | *HCG +ve* | | |
|---|---|---|---|---|---|---|
| *Follow-up (yrs)* | *No. of patients* | *Dead of disease* | *Dead of other cause* | *No. of patients* | *Dead of disease* | *Dead of other cause* |
| 1 | 184 | 10 | 0 | 30 | 3 | 1 |
| 2 | 183 | 14 | 0 | 30 | 7 | 1 |
| 3 | 181 | 17 | 1 | 29 | 7 | 1 |
| 4 | 174 | 18 | 4 | 29 | 7 | 1 |
| 5 | 169 | 19 | 4 | 28 | 7 | 1 |
| 7 | 150 | 21 | 8 | 25 | 7 | 1 |
| 10 | 122 | 22 | 11 | 23 | 7 | 1 |
| 15 | 60 | 22 | 14 | 13 | 7 | 1 |

**Figure 4** Cumulative survival curves of 184 patients with HCG-negative seminomas and 30 with HCG-positive seminomas, stages P1 and P2. The figures in parenthesis indicate the total number of patients in the HCG positive and negative groups followed-up at each period following orchidectomy (details are shown in **Table III**).

### Discussion

The localization of HCG in some seminomas has implications for their histological classification and clinical course. This study is based on a retrospective series of patients who presented between 1958 and 1972. Consequently it was not possible to correlate the presence of HCG-containing cells with circulating levels of HCG, the routine measurement of which is a more recent development. Additionally, treatment and staging procedures improved and categories changed throughout the period during which these tumours were documented. Consequently, the results were related to pathological stage for which information was available and definitions had remained the same, rather than clinical assessment of disease extent. Despite these limitations this study is of clinical relevance because it addresses the question of whether the presence of HCG-containing cells influences survival in the largest series of seminomas available with a long follow-up. The information obtained is of especial value if surveillance rather than active therapy is considered post orchidectomy for patients with clinical stage I disease (Oliver *et al.*, 1983). The identification of HCG-positive cells as a poor prognostic factor may contraindicate withholding treatment in these patients.

Three aspects of HCG-containing cells in seminomas were studied: their morphology, incidence and prognostic significance.

### *Morphology*

Giant cells and multinucleate cells resembling syncytiotrophoblasts are an established morphological feature of some seminomas, the first description being attributed to Chevassu in 1906 (Hedinger *et al.*, 1979), but their definition and incidence in different series have varied (Friedman & Moore, 1946; Dixon & Moore, 1952; Friedman & Pearlman, 1970; Mostofi & Price, 1973; Thackray & Crane, 1976). Some studies include all giant cells except the Langhans cell, whereas others confined themselves to the typical syncytiotrophoblast. Furthermore, the methods of sampling and case selection differ in these studies.

The functional significance of these cells was partially resolved in 1976 when Heyderman and Neville localised HCG in solitary giant cells in germ cell tumours. Since this time HCG in seminomas has been reported variously as occuring exclusively in isolated cells or clusters of cells showing the morphological features of syncytiotrophoblasts (Javadpour *et al.*, 1978; Kurman *et al.*, 1979; Mostofi, 1980), or to be localised commonly in these cells and occasionally seen in seminoma giant cells ("mulberry" cells) (Heyderman, 1978; Bosman *et al.*, 1980) and rarely in "ordinary seminoma cells" (Morgan *et al.*, 1982).

In agreement with previous workers (Heyderman, 1978; Bosman *et al.*, 1980; Morgan *et al.*, 1982; Morinaga *et al.*, 1983) HCG was localised predominantly in typical syncytiotrophoblastic giant cells and in tumour giant cells or mulberry cells and mononuclear seminoma cells. This provides evidence both for a link between different germ cell tumours and the contention that teratomatous elements may develop from seminoma cells.

The relevance of the morphological identification of HCG-producing cells to the histopathologist and clinician is apparent from the finding that seminomas with cells containing HCG form a group with a significantly worse prognosis. Therefore, it is important that these elements can be identified accurately. It is clear in this series that even in a review of H & E sections aimed at assessing the incidence of SGC, these cells will be missed in a high proportion of cases even by an experienced pathologist. These data indicate that the localisation of HCG by immunocytochemistry should form part of the routine assessment of seminoma.

*Incidence*

This is the first large series in which the incidence of HCG-containing cells in seminomas is described, the only other comparable series being that of Bosman *et al.* (1980), who found an incidence of 3/46 (7%). In other publications it is not clear whether all seminomas have been investigated immunocytochemically or whether this technique has been restricted to those tumours in which syncytiotrophoblasts have been seen on H & E stained sections (Hedinger *et al.*, 1979) or those associated with elevated serum levels of HCG (Javadpour *et al.*, 1978).

In 228 seminomas 14.5% were found to contain HCG positive cells. This figure is within the range of published data based on morphological appearances or HCG production. Since, as shown in Table II, a relationship between the number of sections and the HCG positive cells found could not be demonstrated, it is unlikely that sampling error was a problem in this series. However, some bias may have been present in this series for three reasons. Firstly, only tumours from which wax-embedded material was available were examined. Secondly, this material was obtained from a Testicular Tumour Panel and the possibility exists that "unusual" cases, such as those containing SGC, were selectively referred. Thirdly, only four P3 tumours were available, the remaining 224 being of pathological stages P1 and P2.

*Prognostic significance*

The prognostic implication of HCG-containing cells in seminomas has not been established (Javadpour *et al.*, 1978; Hedinger *et al.*, 1979; Mostofi, 1980; Bosman *et al.*, 1980). Differences in mortality rates for patients with and without tumour giant cells in Thackray & Crane's (1976) series did not reach statistical significance. In contrast, Mostofi (1980) found a death rate of 7% in patients whose tumours did not contain syncytiotrophoblastic cells and 28% mortality of those whose tumours did contain such cells.

The presence of syncytiotrophoblastic cells in seminomas has been associated with elevated levels of urinary chorionic gonadotrophin and a worse prognosis (Dixon & Moore, 1952; Wilson & Woodhead, 1972; Maier & Sulak, 1973). However, this approach does not differentiate the influence of disease bulk from the presence of HCG-producing cells. The development of a serum $\beta$-HCG radioimmunoassay provided a more sensitive means for detecting this protein. Elevated levels of serum $\beta$-HCG have been found in 5–33% of patients with seminoma (Cochran *et al.*, 1975; Bartsch *et al.*, 1979; Javadpour, 1980, 1983) but there is still no concensus as to its prognostic significance. Lange *et al.* (1980) suggest that this finding in association with metastatic seminoma is a poor prognostic sign and indicates the use of chemotherapy. A further clinical concern is that elevated HCG associated with seminoma might be related to classical trophoblastic tumour or choriocarcinoma missed by the sampling for histopathology.

The classification of tumours containing syncytiotrophoblasts as seminomas rather than combined tumours is inconsistent, as such cells constitute a teratomatous element. In addition, the natural history of seminomas with HCG-containing cells in this historical study was in two respects similar to that of combined tumours. Firstly, 23% of the patients with HCG-containing tumours were dead of their disease within 2 years of orchidectomy, compared with only 8% of the patients with tumours lacking this feature ($P<0.01$). Secondly, late relapse was not observed in the HCG-positive group, whereas 8/22 of the HCG-negative group died of their disease during the period 2–10 years following orchidectomy. In contrast to seminomas, late relapse is rarely encountered with teratomas. The clinical implication of these data is that seminomas containing HCG-positive cells might benefit more from treatment regimes used for non-seminomatous testicular germ cell tumours than those used for seminomas.

We wish to thank Professor K. Bagshawe for providing the HCG antiserum. The cases reviewed here were obtained from the British Testicular Tumour Registry.

### References

BARTSCH, G., MIKUZ, G., WEISSTEINER, G. & DAXENBICHLER, G. (1979). β-HCG-positive seminome. *Aktuelle Urol.*, **10**, 259.

BOSMAN, F.T., GIARD, R.W.M., NIEUWENHUIJEN KRUSEMAN, A.C., KNIJNENBURG, G. & SPAANDER, R.J. (1980). Human chorionic gonadotrophin and alpha-fetoprotein in testicular germ cell tumours: A retrospective immunohistochemical study. *Histopathology*, **4**, 673.

COCHRAN, J.S., WALSH, P.C., PORTER, J.C., NICHOLSON, T.C., MADDEN, J.D. & PETERS, P.C. (1975). The endocrinology of human chorionic gonadotrophin-secreting testicular tumors: New methods in diagnosis. *J. Urol.*, **114**, 549.

DIXON, F.J. & MOORE, R.A. (1952). Tumors of the Male Sex Organs. In: *Atlas of Tumor Pathology*. Washington DC: Armed Forces Institute of Pathology, Section VIII, Fascicles 31b and 32, p. 57.

FRIEDMAN, N.B. & MOORE, R.A. (1946). Tumors of the testis. A report of 922 cases. *Milit. Surg.*, **99**, 573.

FRIEDMAN, M. & PEARLMAN, A.W. (1970). "Seminoma with trophocarcinoma". A clinical variant of seminoma. *Cancer*, **26**, 46.

HEDINGER, C., VON HOCHSTETTER, A.R. & EGIOFF, B. (1979). Seminoma with syncytiotrophoblastic giant cells. A special form of seminoma. *Virchows Arch. A Pathol. Anat. Histol.*, **383**, 59.

HEYDERMAN, E. (1978). Multiple tissue markers in human malignant testicular tumours. *Scand. J. Immunol.*, **8**, (Suppl. 8) 119.

HEYDERMAN, E. & MUNRO NEVILLE, A. (1976). Syncytiotrophoblasts in malignant testicular tumours. *Lancet*, **ii**, 103.

JAVADPOUR, N. (1980). The role of biologic tumor markers in testicular cancer. *Cancer*, **45**, 1755.

JAVADPOUR, N. (1983). Multiple biochemical tumor markers in seminoma. *Cancer*, **52**, 887.

JAVADPOUR, N., McINTIRE, K.R. & WALDMANN, T.A. (1978). Human chorionic gonadotrophin (HCG) and alpha-fetoprotein (AFP) in sera and tumor cells of patients with testicular seminoma. *Cancer*, **42**, 2768.

KURMAN, R.J., SCARDINO, P.T., McINTIRE, K.R., WALDMANN, T.A., JAVADPOUR, N. & NORRIS, H.J. (1979). Malignant germ cell tumors of the ovary and testis. An immunohistological study of 69 cases. *Ann. Clin. Lab. Sci.*, **9**, 462.

LANGE, P.H., NOCHOMOVITZ, L.E., ROSAI, J. & 15 others. (1980). Serum alpha-fetoprotein and human chorionic gonadotropin in patients with seminoma. *J. Urol.*, **124**, 472.

MAIER, J.G. & SULAK, M.H. (1973). Radiation therapy in malignant testis tumors, Part II, Carcinoma. *Cancer*, **32**, 1217.

MORGAN, D.A.L., CAILLAUD, J.M., BELLET, D. & ESCHWEGE, F. (1982). Gonadotrophin-producing seminoma: A distinct category of germ cell neoplasm. *Clin. Radiol.*, **33**, 149.

MORINAGA, S., OJIMA, M. & SASANO, N. (1983). Human chorionic gonadotropin and alpha-fetoprotein in testicular germ cell tumors. *Cancer*, **52**, 1281.

MOSTOFI, F.K. (1980). Pathology of germ cell tumors of testis. A progress report. *Cancer*, **45**, 1735.

MOSTOFI, F.K. & PRICE, E.B. (1973). Tumors of the male genital system. In: *Atlas of Tumor Pathology*. (Ed. Firminger), Washington DC: Armed Forces Institute of Pathology, Second Series, Fascicle 8, p. 24.

OLIVER, R.T.D., HOPE-STONE, H.F. & BLANDY, J.P. (1983). Justification of the use of surveillance in the management of stage 1 germ cell tumours of the testis. *Br. J. Urol.*, **55**, 760.

PECKHAM, M.J. (1981). Seminoma testis. In: *The Management of Testicular Tumours*. (Ed. Peckham), London: Edward Arnold, p. 134.

PETO, R. & PIKE, M.C. (1973). Conservatism of the approximation in the log rank test for survival data or tumour incidence data. *Biometrics*, **29**, 579.

PUGH, R.C.B. (1976). Combined tumours. In: *Pathology of the Testis*. (Ed. Pugh), Oxford: Blackwell Sci. Publ., p. 245.

STERNBERGER, L.A., HARDY, P.H., CUCULIS, J.I. & MEYER, H.C. (1970). The unlabeled antibody-enzyme method of immunohistochemistry. Preparation and properties of soluble antigen-antibody complex (horseradish peroxidase-antihorseradish peroxidase) and its use in the identification of spirochetes. *J. Histochem. Cytochem.*, **18**, 315.

THACKRAY, A.C. & CRANE, W.A.J. (1976). Seminoma. In: *Pathology of the Testis*. (Ed. Pugh), Oxford: Blackwell Sci. Publ., pp. 164–198.

WILSON, J.M. & WOODHEAD, D.M. (1972). Prognostic and therapeutic implications of urinary gonadotropin levels in the management of testicular neoplasia. *J. Urol.*, **108**, 754.