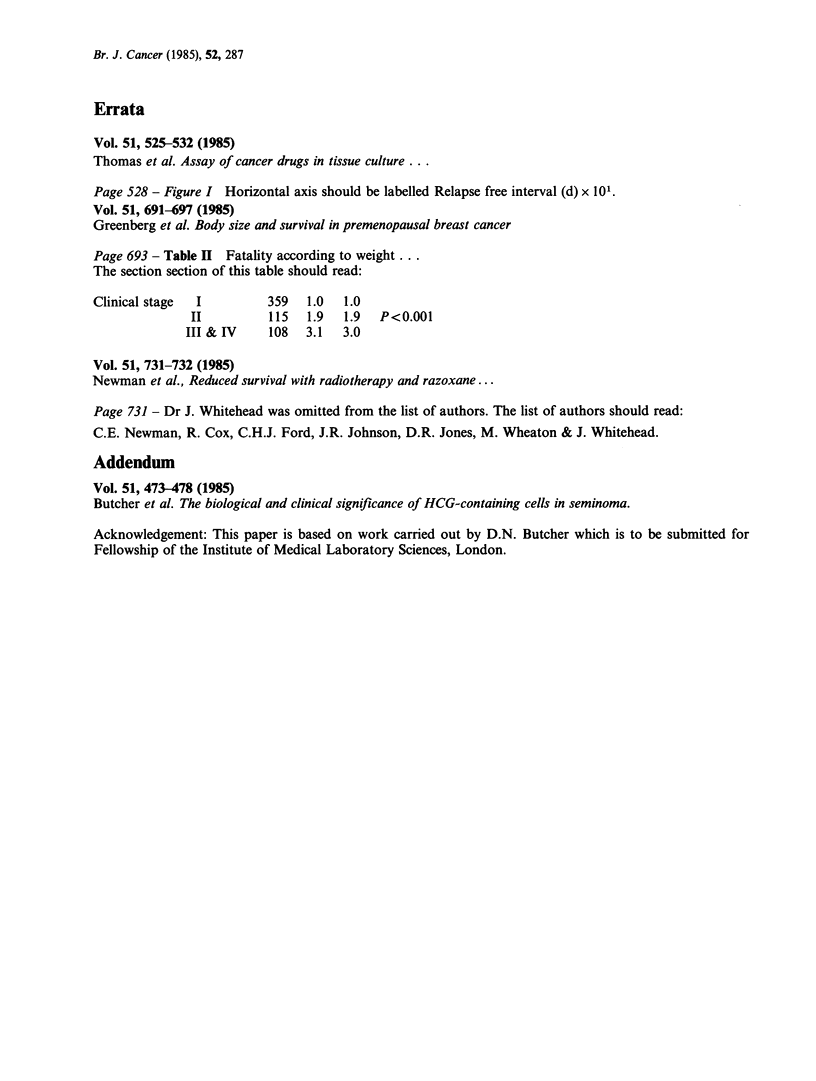# Addendum

**Published:** 1985-08

**Authors:** 


### Addendum

**Vol. 51, 473–478 (1985)**
Butcher *et al. The biological and clinical significance of HCG-containing cells in seminoma.*

Acknowledgement: This paper is based on work carried out by D.N. Butcher which is to be submitted for Fellowship of the Institute of Medical Laboratory Sciences, London.